# Magnetic resonance manifestation of diffuse osteopenia in a cat with presumed nutritional secondary hyperparathyroidism, hypocobalaminaemia and thiamine deficiency

**DOI:** 10.1111/jsap.13815

**Published:** 2025-01-15

**Authors:** A. Skarbek, C.‐G. Danciu, J. Fenn, J. Klever

**Affiliations:** ^1^ The Department of Small Animal DiagnosticImaging Queen Mother Hospital for Animals Hatfield UK

**FIG 1:**
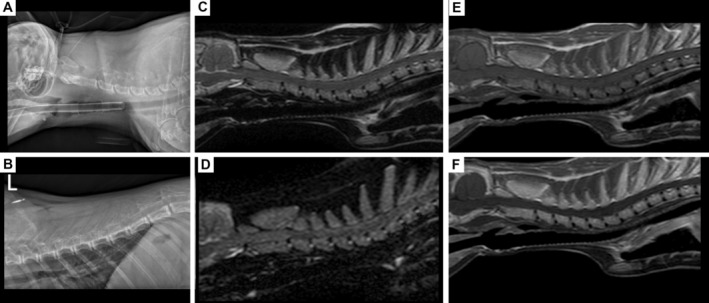
Lateral radiograph of the cervical and thoracic vertebral column showing markedly reduced mineral opacity and cortical thinning in the axial skeleton (A, B). Magnetic resonance imaging of the cervical vertebral column demonstrating the marked hyperintensity of the included skeleton in T2w (C), STIR (D) and T1w (E) sequences. After gadolinium administration, a strong, diffuse contrast enhancement of the bones is demonstrated in the sagittal post‐contrast T1w sequence (F).

A 1‐year‐old male entire Bengal cat presented for weakness and progressive gait abnormality over 5 days. The cat was fed raw or cooked chicken since a kitten and was diagnosed with left‐sided femoral neck fracture after a fall several months prior. Neurological examination revealed appropriate mentation, intermittent intention tremor, non‐ambulatory tetraparesis with generalised proprioceptive ataxia, reduced postural reactions, intact spinal reflexes in all limbs and unremarkable cranial nerves. Neuroanatomical localisation was C1‐5 spinal cord segments and/or cerebellum. Haematology revealed neutrophilia [23.88 × 109/L, reference interval (RI): 2.50 to 12.50 × 109/L]. Serum biochemistry revealed hypernatraemia (160.0 mmoL/L, RI: 145.0 to 157.0 mmoL/L), hypocalcaemia (1.96 mmoL/L, RI: 2.11 to 2.90 mmoL/L), hypercholesterolaemia (5.2 mmoL/L, RI: 2.2 to 4.0 mmoL/L), increased alanine transaminase (159 U/L, RI: 5 to 60 U/L) and hypocobalaminaemia (154.0 ng/L, RI: >200.0 ng/L). The radiographs of the skull, vertebral column and thoracic limbs revealed diffuse osteopenia of the axial and appendicular skeleton, including decreased mineral opacity, cortical thinning and double cortical lign in the left radius. The included bones were enlarged and showed abnormal shape, including enlarged dens protruding into the vertebral canal. MRI of the head and cervical region demonstrated unremarkable brain, marked diffusely increased T2‐weighted (T2w), T1w, short tau inversion recover (STIR) signal intensity, loss of cortical and trabecular bone distinction and strongly contrast enhancing axial skeleton. The radiographic and MRI features were considered secondary to osteopenia and fibrous osteodystrophy related to nutritional hyperparathyroidism. Due to the cerebellar signs and dietary history, thiamine deficiency was suspected alongside the hypocobalaminaemia. A balanced diet, cobalamin and thiamine supplementation led to complete clinical recovery. Decreased cellular marrow components, premature red‐to‐yellow conversion and increased fat content could elicit T1w skeletal hyperintensity. Further MRI signal changes including contrast enhancement are not fully understood; however, incomplete red‐to‐yellow conversion and serous atrophy of the bone marrow are suspected (Fig 1).

